# Is it okay to feel this way? Exploring the joint effect of emotional experiences and expectations on life satisfaction

**DOI:** 10.3389/fpsyg.2024.1305812

**Published:** 2024-02-28

**Authors:** June Chun Yeung, Marta Roczniewska, Kuba Krys

**Affiliations:** ^1^Institute of Psychology, Polish Academy of Sciences, Warsaw, Poland; ^2^Institute of Psychology, SWPS University, Sopot, Poland; ^3^Medical Management Centre, Karolinska Institutet, Stockholm, Sweden

**Keywords:** emotional experience, societal expectation, emotional norms, life satisfaction, response surface analysis

## Abstract

This research investigates the joint effect of individual emotional experiences and societal expectations on life satisfaction. Inspired by the Affect Valuation Theory and Self-Discrepancy Theory, we explored how discrepancies between actual emotional experiences and what society believes we “ought” to feel are linked with life satisfaction. A total of 301 U.S. online participants rated their emotional experiences and societal expectations for emotions, along with measures of life satisfaction. Response surface analyses were used to assess the effect of emotional experience-norm congruence on life satisfaction. Findings revealed that the highest life satisfaction reported by individuals infrequently experiencing negative emotions but perceiving high societal expectations for these emotions, while congruence effects were not supported. These findings suggest the potential benefits of a societal shift toward greater acceptance of a wider range of negative emotions. The study may potentially stimulate interventions to enhance individuals' life satisfaction by reconsidering societal beliefs about emotions.

## 1 Introduction

Our emotional experiences are embedded in societal norms, creating a complicated environment that influences personal wellbeing, including its key component—life satisfaction. The Affect Valuation Theory (Tsai, 2007) and the Self-Discrepancy Theory (Higgins, 1987) have contributed valuable insights into the understanding of these dynamics. These theories highlight the significance of discrepancies between various emotions and self-concept, and their consequential effects on individual wellbeing. Nonetheless, there exists limited knowledge regarding the specific effects of the alignment between emotional experiences and societal expectations on individual wellbeing. Our objective is to investigate these dynamics and their effects on life satisfaction, one of the key facets of subjective wellbeing.

The Affect Valuation Theory, as developed by Tsai (2007), addresses the complexities surrounding emotions, particularly the differences between individuals' emotional experiences (actual affect) and the emotions that they are value and strive to experience (ideal affect). According to the study by Tsai et al. (2006), cultural structures play a pivotal role in shaping our ideal emotional states. Different cultures have varying preferences for prioritizing positive emotional states. For instance, Western cultures, such as the United States, often prioritize high-arousal positive states like excitement, while East Asian cultures, like Hong Kong and Asian Americans, place more emphasis on low-arousal positive states such as calmness (Tsai et al., 2006). The study by De Almeida and Uchida (2021) further illustrates that in Latin American cultures, there is a tendency to value high-arousal positive emotions, whereas East Asian cultures favor low-arousal positive emotions. The Affect Valuation Theory also provides insight into the influence of cultural norms on individuals' emotional desires and, consequently, their overall wellbeing. Research conducted through Affect Valuation Theory has revealed that disparities between an individual's current emotional state and their desired emotional state can have a substantial impact on their wellbeing (Scheibe et al., 2013).

In terms of theoretical frameworks concerning discrepancy, the Self-Discrepancy Theory (Higgins, 1987) presents a comprehensive viewpoint on the internalized standards of persons. According to this theory, individuals are considered to act within three fundamental aspects of the self: the “actual self” (representing one's current state of being), the “ought self” (reflecting social or personal expectations for one's identity), and the “ideal self” (representing one's aspirations and desired identity). It is hypothesized that disparities or misalignments among these areas can result in different emotional consequences. For example, according to Higgins (1987), when there is a difference between an individual's actual self and their ought self, it can lead to the experience of guilt or worry. On the other hand, when there is a disparity between an individual's actual self and their ideal self, it can result in feelings of dejection or disappointment. Furthermore, some research on cultural variations in self-regulation highlights how different cultural contexts can influence the “ought self,” affecting individuals' emotional regulation and experiences (Trommsdorff, 2009). The “ought self,” which is strongly shaped by the contextual norms and expectations, holds particular significance in shaping emotional experiences.

The Affect Valuation Theory offers significant insights into the disparity that exists between individuals' experienced emotions and their desired or ideal feelings. In particular, Tsai (2007) examined the impact of cultural nuances on our emotional standards, which subsequently influences our interpretation of individual experiences. In contrast, the Self-Discrepancy Theory places emphasis on the emotional consequences that arise as a result of disparities between our present, desired, and idealized selves (Higgins, 1987). The ought-self represents the attributes that individuals believe they should possess, often influenced by societal expectations or obligations. While the impact of the actual-ideal discrepancy in feelings on wellbeing has been documented (e.g., Scheibe et al., 2013; Schlechter et al., 2022), to our knowledge, the effects of the actual-ought discrepancy in emotion remain unexplored (cf. Higgins et al., 1997 for the general actual-ought discrepancy effect).

The existence of this study gap does not imply that the examination of societal views of emotions on subjective wellbeing lacks significance in the scientific literature. Indeed, the act of exclusively esteeming happy emotions while diminishing unpleasant emotions might paradoxically result in feelings of sadness and impose societal drawbacks (Yeung and Lun, 2016, 2021). The avoidance of negative emotions has been found to have a detrimental impact on wellbeing (Bastian et al., 2012; Humphrey et al., 2022). Therefore, it is crucial to acknowledge the possible impact of “ought feelings” on an individual's subjective wellbeing (in our study, which is manifested by life satisfaction), and the way in which these expectations interact with actual emotional experiences can significantly shape it.

In order to examine the joint effect of experienced and expected emotions, a potential perspective to consider is the congruence or fit models (i.e., Chatman, 1989), which propose that when there is alignment or fit between multiple entities (e.g., person-job, person-organization, person-group fit), may lead to favorable outcomes (Kristof-Brown et al., 2005). Conversely, when there is misalignment, it can result in negative consequences (Ostroff, 2012). Within the scope of our research, the alignment between an individual's emotional encounters and the prevailing society norms can be examined through the use of this theoretical framework. If the principles of the fit model were to be fully applied to our study, one could hypothesize that a consistent alignment between an individual's emotional experiences and societal expectations around those emotions might lead to increased life satisfaction. Such congruence, where internal emotional states align with external social norms, could foster a sense of coherence, validation, and belonging. On the other hand, a misalignment, where genuine emotions diverge from societal norms, could potentially evoke feelings of isolation, incompetence, or anxiety.

Nevertheless, although the fundamental principles of the congruence model offer a persuasive framework, there exist intricacies in real-world scenarios that may pose obstacles to its direct implementation. For example, the inherent characteristics of emotions, such as their dynamic nature and subjective expressions, may render perfect alignment or congruence not always preferable. According to Gross and John (2003), there are instances where rigidly conforming to society norms may hinder the authentic expression of emotions, resulting in psychological distress. Furthermore, it is important to acknowledge the fluidity of social standards, as they undergo changes through time, across different cultures, and within various circumstances. This implies that the definition of “fit” might be temporary and subject to variation (Edwards, 2008). In the present investigation, it is possible that the congruence effect may not manifest in the conceptual sense. It is challenging to imagine the existence of an individual who consistently encounters bad emotional experiences while maintaining a high level of wellbeing, despite societal expectations that dictate otherwise. Therefore, although congruence models provide useful insights into the possible alignment between individual emotions and societal expectation, it is crucial to approach its implications with subtlety, recognizing the diverse circumstances that may impact the dynamics of this interaction in real-world scenarios.

To investigate the complex interplay between emotional experiences and societal expectations, we employed a rigorous analytical methodology. Traditional approaches for differences studies employed algebraic difference or residual scores to demonstrate the gaps between two constructs. While these methodologies have offered foundational understanding, they are not exempt from limitations. One of the main drawbacks involved with the utilization of difference score approaches is the possible risk of information loss (Edwards, 2002). While the algebraic differences of (2 − 1) and (9 − 8) both result in a value of 1, their conceptual implications in the context of emotional expression and expectation congruence are significantly distinct. Consider the expression (2 − 1), where the societal expectation for an emotion is 2 and the actual emotional experience is 1. This represents a minor discrepancy within a low-level emotional profile, suggesting that both the emotion and its societal expectation are relatively trivial. In contrast, the expression (9 − 8), where the societal expectation is 9 and the actual experience is 8, indicates a minor discrepancy within a high-level emotional profile. This scenario implies a strong emotion being experienced that nearly matches an equally high societal expectation. This distinction demonstrates how differences of the same numerical value can have varying implications depending on the levels of emotional intensity and societal expectations involved, potentially influencing individual life satisfaction in different ways.

In contrast to conventional methodologies, polynomial regression with response surface analysis (RSA) offers a more accurate and comprehensive approach. As highlighted by Humberg et al. (2018), this is a statistical technique that can understand and interpret the relation between two independent variables and the dependent variable in a three-dimensional space, by considering both the direction and magnitude of discrepancies between these variables. This surface is a graphical representation that allows us to visualize the relation between these variables and their combination effects. This model considers not just the linear relation but also the quadratic and interaction terms, which are crucial when the relation between variables is not merely additive (Edwards, 2002).

In practice, RSA involves fitting a polynomial regression model to the data, which is essentially an extension of linear regression. It allows for the examination of nonlinear relations by considering higher-order terms (squares and products of the predictor variables). RSA specifically investigates congruence through the line where both predictors are equal (the line of congruence), and incongruence through the line where they are opposite (the line of incongruence) (Edwards, 2002). The line of congruence represents scenarios where both variables increase together, indicating a harmonic relationship, while the line of incongruence illustrates scenarios where one variable increases as the other decreases, suggesting a discordant relationship. This distinction is pivotal for understanding the dynamics between variables and their impact on the outcome of interest. Hence, we can test whether individuals experience the highest life satisfaction when their emotional experiences perfectly match societal expectations, or if there's a particular combination of variables that leads to optimal outcomes (Barranti et al., 2017). RSA also provides a visual representation of this relation, usually through 3D plots, which illustrate the outcome variable as a function of the two predictor variables (Barranti et al., 2017). This visual aid is instrumental in interpreting the interaction effects and in understanding the complexity of the data. Thus, we may gain a deeper and more accurate understanding of the dynamics between emotional experiences and expectation on life satisfaction.

In summary, our objective is to explore a comparatively unexplored domain of the actual-ought emotional discrepancy, comprehend its potential effects on life satisfaction, and consequently, potentially inform future therapies designed to improve individual life satisfaction. The utilization of response surface analysis in our technique enables us to investigate the combined impact of variables while minimizing the loss of data information. The present study holds the potential to establish a basis for broader discussions concerning the social perspectives and evaluations of emotions, as well as the potential for recalibrating these perceptions to enhance people's life satisfaction, and in consequence the overall societal wellbeing.

## 2 Method

### 2.1 Participants

The research had a sample size of 301 individuals residing in the United States who willingly participated in the study through the Prolific platform. The study population consisted of 145 female participants and 149 male participants, ranging in age from 18 to 50 years. The mean age was 34.41, with a standard deviation of 7.91. In order to enhance the representativeness of the online population in the United States, the selection of participants was conducted from a non-student, community sample.

### 2.2 Measures

#### 2.2.1 Emotional experience and social expectations for emotions

In this study, participants were asked to report their emotional experiences and their perceived social expectations for emotions. The scale for emotional experiences was adapted from Krys et al. (2022), selecting a mix of 12 distinct emotions based on the factor loadings in the original study. These emotions were categorized into six positive (grateful, excited, peaceful, relaxed, in love, enthusiastic) and six negative emotions (fearful, angry, sad, ashamed, depressed, dull). Similarly, the items for perceived societal expectations were inspired and adapted from Bastian et al. (2012). Participants reported both the frequency of their emotional experiences (e.g., “your frequency of experience: grateful”) and their perception of societal expectations for each emotion (e.g., “Your society expects you should feel: grateful”). The scale's responses ranged from 1 to 9 (1 = *none in a week*, 5 = *once a day*, 9 = *all the time*). The reliability of this scale was substantiated by Cronbach's alpha, with scores ranging from 0.79 for Positive Emotional Experiences to 0.88 for Expectation for Negative Emotions (see Table 1).

**Table 1:** Descriptive statistics and correlations among focal variables.

**Variables**	***M***	***SD***	**α**	**1**	**2**	**3**	**4**
1. Life satisfaction	2.45	1.10	0.93				
2. Positive emotional experiences	4.07	1.44	0.79	0.54^**^			
3. Expectation for positive emotion	5.10	1.87	0.87	0.06	0.24^**^		
4. Negative emotional experiences	3.38	1.63	0.87	−0.50^**^	−0.30^**^	0.14^*^	
5. Expectation for negative emotions	2.54	1.55	0.88	0.03	0.27^**^	−0.20^**^	0.20^**^

^***^p < 0.001.

^**^p < 0.01.

^*^p < 0.05.

#### 2.2.2 Life satisfaction

Life satisfaction, a key component of subjective wellbeing, was assessed in our study using the Satisfaction with Life Scale (Diener et al., 1985). Recognized as a reliable measure in diverse sociocultural contexts, this scale is instrumental in evaluating a happiness-related aspect of subjective wellbeing. Sample items from this scale include statements like “In most ways my life is close to my ideal” and “I am satisfied with my life.” The scale demonstrated high reliability with a Cronbach's alpha of 0.93.

### 2.3 Procedure

Upon enrollment via the Prolific platform, participants were directed to Qualtrics online portal. They were briefed about the study's objectives and the confidentiality of their responses. After providing informed consent, participants responded to the above-listed scales and demographic questions.

### 2.4 Data analysis

Descriptive statistics and correlations among focal variables were assessed. Paired-sample *t*-test were employed to examine the discrepancies between emotional experience and social expectation for positive and negative emotions. Polynomial regressions with response surface analyses [Shanock et al. (2010); Edwards and Parry (1993), RSA package: Schönbrodt and Humberg (2021)] was conducted to determine the relation between experience-norm congruence for positive and negative emotions and life satisfaction. All analyses were conducted in R (R Core Team, 2022, 4.2.2), with anonymised data and script available online at: https://osf.io/h683g/.

## 3 Results

Descriptive statistics, including *M*, *SD*, and correlations between all the focal variables are provided in the Table 1. In general, individuals perceived they should experience positive emotions moderately more frequently than they actually experienced (*t*[300] = 8.62, *p* < 0.001, *d* = 0.50) and experience negative emotions moderately less frequently than they actually experienced (*t*[300] = −7.30, *p* < 0.001, *d* = −0.42).

**Figure 1:**
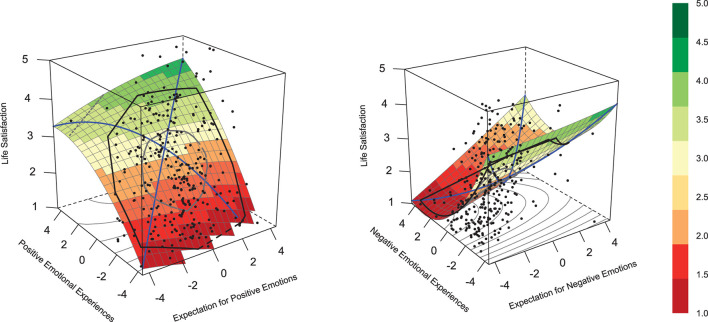
Surface plots display experience-norm congruence for positive **(left)** and negative **(right)** emotions. The experience-norm congruence is the combination between emotional experience (*y* axis) and expectation for emotions (*x* axis) on life satisfaction (*z* axis).

Two polynomial regressions were estimated and visualized in three-dimensional surface plots. The explained variance of the global models, regression coefficients, principal axes, and surface tests estimates can be found in Table 2. A detailed description of all the regression parameters and surface parameters can be found in Supplementary material. The parameters *a*1 and *a*2 correspond to the slope and curvature along the line of congruence, respectively. In contrast, parameters *a*3 and *a*4 are associated with the slope and curvature along the line of incongruence (Edwards and Parry, 1993). Figure 1 depict the estimated regression model for life satisfaction by both experience-norm congruence for positive and negative emotions.

**Table 2:** Response surface results for the effect of experience-norm congruence on life satisfaction.

		**Estimated regression models**			
		**Positive emotions**		**Negative emotions**	
		**Estimates (SE)**	**CI**	**Estimates (SE)**	**CI**
**Standardized regression coefficients for models**					
	*b*_0_	2.87 (0.08)^***^	2.71, 3.02	2.19 (0.12)^***^	1.96, 2.42
	*b*_1_	−0.01 (0.04)	−0.08, 0.06	0.08 (0.06)	−0.04, 0.20
	*b*_2_	0.38 (0.05)^***^	0.29, 0.48	−0.17 (0.07)^*^	−0.30, −0.04
	*b*_3_	−0.001 (0.01)	−0.03, 0.02	−0.04 (0.02)^*^	−0.07, −0.01
	*b*_4_	0.04 (0.02)^*^	0.01, 0.07	0.04 (0.02)^†^	−0.001, 0.09
	*b*_5_	−0.03 (0.02)	−0.07, 0.01	0.06 (0.01)^***^	0.04, 0.09
**Position of first principal axis**					
	*p*_10_	5.16 (2.66)†	−0.05, 10.37	−6.55 (6.35)	−19.00, 5.90
	*p*_11_	0.53 (0.33)	−0.12, 1.18	5.05 (2.38)^*^	0.40, 9.71
**Shape of surface along lines**					
LOC	*a*_1_	0.37 (0.06)^***^	0.25, 0.49	−0.09 (0.09)	−0.27, 0.10
	*a*_2_	0.01 (0.03)	−0.04, 0.07	0.07 (0.03)^*^	0.01, 0.12
LOIC	*a*_3_	−0.39 (0.06)^***^	−0.51, −0.28	0.25 (0.09)^**^	0.08, 0.42
	*a*_4_	−0.07 (0.02)^**^	−0.11, −0.02	−0.02 (0.03)	−0.08, 0.05

^***^p < 0.001.

^**^p < 0.01.

^*^p < 0.05. ^†^*p* < 0.10.

Outcome variables: life satisfaction. LOC, line of congruence; LOIC, line of incongruence. Polynomial regression model: $Z=b_{0}+b_{1}X+b_{2}Y+b_{3}X^{2}+b_{4}XY+b_{5}Y^{2}$; *X* = expectation for emotions, *Y* = emotional experience. $R_{positive}^{2}=30.8\%$, $R_{negative}^{2}=34.8\%$.

We firstly examined the effect of congruence by Humberg et al. (2019)'s checklist. In all the analyses, at least one condition was violated and the RSA contradicted a congruence effect, indicating that those who simply feel fit their perceived expectations were not those who have the highest level of life satisfaction. Although there were no effects of congruence, the result can be interpreted (see Breetzke and Wild, 2022, for a similar practice). Especially the linear, curvilinear, possible interaction effects, and the direct interpretation of the potential effects of emotional fit on life satisfaction are of our major interests.

Linear effects of the emotional experience were the most salient effects in our data. Specifically, the higher frequency of positive emotions was associated with a greater level of life satisfaction (*b*_2_ = 0.38, *SE* = 0.05, *p* < 0.001), indicating that more frequent positive emotions contribute to higher life satisfaction. Conversely, a higher frequency of negative emotions was associated with a lower level of life satisfaction (*b*_2_ = −0.17, *SE* = 0.07, *p* = 0.01). Some of the higher-order terms were significant, resulting in non-flat surfaces, which indicates a more complex relation than a simple linear one.

The interaction terms for positive emotions (*b*_4_ = 0.04, SE = 0.02, *p* = 0.01) was significant, indicating a joint effect of individual emotional experience and perceived societal expectation on life satisfaction. As for the surface parameters, all instances of *a*_3_ (representing the slope of the line of incongruence, LOIC) were significant (positive emotions: *a*_3_ = −0.39, SE = 0.06, *p* < 0.001; negative emotions: *a*_3_ = 0.25, SE = 0.09, *p* = 0.004), indicating a joint influence of individual emotional experience and perceived societal expectation on life satisfaction. This means the relation strength between incongruence and the outcome depends on the specific discrepancy direction. The lowest levels of life satisfaction were reported by individuals frequently experiencing negative emotions alongside low perceived societal expectations for such emotions, whereas the highest life satisfaction was reported by those infrequently experiencing negative emotions but perceiving high societal expectations for these emotions.

Additionally, along the line of congruence for negative emotions, although the linear effect was not significant (*a*_1_ = −0.09, SE = 0.09, *p* = 0.35), the analysis revealed a significant curvature effect (*a*_2_ = 0.07, SE = 0.03, *p* = 0.02). This finding points to a non-linear relation between the congruence of negative emotional experiences and expectations, and life satisfaction. However, the high congruence condition of negative emotions (high expectations with high experiences) was found to have an insufficient number of instances to robustly support this curvature effect. This limitation indicates that the curvature effect may not precisely represent the pattern for this specific situation. The analysis continues to suggest a non-linear relation; yet, the clarity of this relation for scenarios with high congruence of negative emotions is less certain. Therefore, caution should be exercised when interpreting the curvature effect for high congruence of negative emotions.

## 4 Discussion

Through our empirical inquiry into the intricate relation between individual emotional experiences and societal expectations, we have discovered the effects on life satisfaction, the happiness-related key component of subjective wellbeing. In contrast to the congruence effects, our findings revealed that individuals who reported rare experiences of negative emotions, but believed high societal expectations for such feelings, reported the highest levels of life satisfaction. This elucidates the possible benefits of societies embracing a wider range of negative emotions.

The present study expands the scope of emotion research by delving into the realm of the actual-ought emotional difference, which has received less attention compared to the examined actual-ideal emotional discrepancy. This study combines elements from the Affect Valuation Theory (Tsai, 2007) and the Self-Discrepancy Theory (Higgins, 1987) to explore the intersection between emotional norms and actual emotional experiences. By integrating these two paradigms, this investigation aims to deepen our understanding of the complex relation between these factors. Significantly, the deviation observed from the potential results predicted by congruence models (e.g., Chatman, 1989)—which suggest that congruence between internal experiences and external expectations generally enhances wellbeing—indicates the necessity of revisiting the general applicability of these models, particularly in relation to emotional experiences and expectations. Moreover, the study revealed that the most significant impacts observed were linear in nature, specifically related to emotional experiences. The significance of good emotions in promoting wellbeing has been acknowledged (Fredrickson, 2001). It has been continuously observed that individuals who frequently experience positive emotions, regardless of cultural norms, tend to report higher levels of life satisfaction.

From a practical standpoint, the study highlights the adverse consequences of societal expectation that may marginalize or diminish the significance of unpleasant emotions. It suggests there are advantages to be gained from a society transition that embraces a wider range of negative emotions. The results of our study suggest that individuals may enjoy an moderate level of life satisfaction when they perceive that their negative emotional experiences are acknowledged and accepted by society. This probable explanation is consistent with the observed phenomenon of those who report the highest levels of life satisfaction being those who infrequently experience negative emotions, yet perceive a high level of society expectations around these emotions. When there is societal acceptance or even an expectation for individuals to experience negativity, it can potentially enhance their life satisfaction, particularly for those who frequently encounter such negative feelings. This notion is aligned with the research of Ford et al. (2018), who found that individuals who accept their negative emotions and thoughts exhibit better psychological health. Additionally, the study by Dejonckheere et al. (2022) indicates that perceiving societal pressure to be happy, particularly in nations with high happiness indices, is linked to poorer wellbeing. These findings highlight the complex interplay between societal expectation on emotions and individual wellbeing, suggesting that the acknowledgment of negative emotions in society can have beneficial effects. This comprehension holds significant implications for therapeutic strategies, since therapists and counselors possess a broader awareness of the emotional dynamics that arise from society norms and expectations. Moreover, at a social level, it calls for the implementation of campaigns or interventions designed to reshape public attitudes toward emotions, hence facilitating the development of a more inclusive and empathic society (Bastian et al., 2012; Yeung and Lun, 2021; Humphrey et al., 2022).

In our discussion of limitations, it is crucial to acknowledge the challenges encountered in supporting the curvature effect for high congruence of negative emotions due to an insufficient number of observations. Future research could benefit from larger sample sizes or more targeted sampling strategies to more accurately capture and understand the effect of emotional congruence effects on life satisfaction. It is also important to approach this interpretation with caution because individuals' experiences and perceptions of emotions vary significantly across various cultural contexts. Although the current exploratory findings offer valuable insights, it is important to replicate them in order to strengthen the reliability and validity of the research conclusions. In keeping with the notion of Constraints on Generality as proposed by Simons et al. (2017), this analysis acknowledges the limits related to the sample. The present sample, which comprises only of online participants from the United States, imposes limitations on the extent to which the findings can be generalized. In order to adequately address the variances in emotional norms and expectations across different cultures and in a culturally sensitive way (Badaan and Choucair, 2023; Thomas and Markus, 2023), it is crucial to incorporate multiple cultural contexts into the future research agenda.

Furthermore, it is important to acknowledge that this study provides valuable insights into the complex interplay between emotional experiences and expectation. However, it is crucial to recognize and take into account many significant limitations associated with this research. Initially, our focus was focused on the examination of “ought emotions” while not thoroughly investigating the specifics and potential impacts of “ideal emotions.” Although these two concepts are separate in nature, it is highly probable that they have a mutual influence on each other. Therefore, excluding one of them from our research could have resulted in the exclusion of useful discoveries. Moreover, if we conceptualize “ought emotions” as a type of injunctive norm, it is possible to think that other associated constructs, such as the emotional context in which individuals are immersed (representing the descriptive norms of emotions), could have a substantial influence on the subjective wellbeing of individuals (Krys et al., 2022). Although our study did not explore these areas, the possible interaction between emotional environment, injunctive norms, and their combined influence on life satisfaction could be significant. Further investigation is warranted to explore the interconnectedness of these notions and gain a more comprehensive understanding of how social and interpersonal emotional norms and contexts interact and influence individual life satisfaction. It is important to acknowledge that life satisfaction is only one of the components of subjective wellbeing (Krys et al., 2024). Future studies are needed to understand the consequences of interplay between emotional experiences and expectation on other—less happiness-related—components of subjective wellbeing, like a sense of meaning, harmony, or a spirituality.

### 4.1 Conclusion

The present study enhances our comprehension of the complex dynamics between emotional experiences, expectations, and life satisfaction. Our findings challenge the one-sided perspectives that advocate for maximizing positive emotions and minimizing negative ones from popular beliefs, by highlighting the potential advantages of emotional validation and the acknowledgment of negative emotions. In order to foster an expanded understanding of emotions and wellbeing, it is our aim that our discoveries serve as an inspiration to continue research in this domain and encourage contemplation on our collective societal perspectives on emotions. By adopting this approach, it is possible to foster a societal environment in which the promotion of accepting negative emotions over sticking to a predetermined set of feelings deemed expected.

## Data availability statement

The original contributions presented in the study are publicly available. This data can be found at: https://osf.io/h683g/.

## Ethics statement

The study was approved by the Research Ethics Committee of the Institute of Psychology of the Polish Academy of Science (approval no. #18/VII/2022). The studies were conducted in accordance with the local legislation and institutional requirements. The participants provided their informed consent to participate in this study.

## Author contributions

JY: Conceptualization, Formal analysis, Investigation, Methodology, Visualization, Writing—original draft, Writing—review & editing. MR: Funding acquisition, Methodology, Writing—review & editing. KK: Funding acquisition, Supervision, Writing—review & editing.
