# Supplementary material for: Is it okay to feel this way? Exploring the joint effect of emotional experiences and expectations on life satisfaction
Source: Front Psychol. 2024 Feb 28;15:1305812. doi: 10.3389/fpsyg.2024.1305812 (PMC10936560; doi:10.3389/fpsyg.2024.1305812)
Supplement: Supplementary file 1 [file Data_Sheet_1.pdf]

frontiers

Is it okay to feel this way? Exploring the joint effect of emotional experiences and expectations on life satisfaction – Supplementary Material

This Supplementary Material is prepared to provide detailed explanations of the statistical results and terms used in our study.

1 POLYNOMIAL REGRESSION OVERVIEW

This section introduces the basics of polynomial regression, a statistical technique used to model the relation between a dependent variable and one or more independent variables. Unlike simple linear regression, polynomial regression allows for a curved line to fit the data points, providing a more nuanced understanding of complex relation between the variables. In our study, we applied polynomial regression with response surface analysis to explore the intricate dynamics between emotional experiences, societal expectations, and life satisfaction. This method is particularly suited for our data, as it accommodates non-linear patterns that are often observed in psychological research, offering insights that go beyond the scope of traditional linear models.

The polynomial regression model with response surface analysis (RSA) used in our study is expressed as:

$$Z = b_0 + b_1X + b_2Y + b_3X^2 + b_4XY + b_5Y^2 \quad \text{(S1)}$$

where Z represents the outcome variable (life satisfaction), X is the expectation for emotions, and Y is the emotional experience. The terms $b_0, b_1, ..., b_5$ are the standardized regression coefficients. To apply this equation to our study:

for Positive Emotions:

$$LS = b_0 + b_1 \times xpect_{pos} + b_2 \times xperi_{pos} + b_3 \times xpect^2_{pos} + b_4 \times xpect_{pos} \times xperi_{pos} + b_5 \times xperi^2_{pos} \quad \text{(S2)}$$

for Negative Emotions:

$$LS = b_0 + b_1 \times xpect_{neg} + b_2 \times xperi_{neg} + b_3 \times xpect^2_{neg} + b_4 \times xpect_{neg} \times xperi_{neg} + b_5 \times xperi^2_{neg} \quad \text{(S3)}$$

In these equations, LS represents the outcome variable of life satisfaction as influenced by positive and negative emotions, respectively. The terms $xpect_{pos}$, $xperi_{pos}$, $xpect_{neg}$, and $xperi_{neg}$ represent specific variables for expectations and experiences of positive and negative emotions. The coefficients $b_0, b_1, ..., b_5$ are as defined in the regression model which will be described and explained as below.

2 REGRESSION PARAMETERS

In the polynomial regression model, each coefficient plays a specific role in shaping the relation between the independent variables (emotional expectations and experiences) and the dependent variable (life satisfaction). Below, we provide a detailed explanation of these coefficients:

1. b_0 **- Intercept:** This coefficient represents the baseline level of life satisfaction when both emotional expectations and experiences are at zero. It provides a starting point for the regression model.
2. b_1 **- Linear Effect of Emotional Expectations:** This coefficient b_1 measures the direct effect of emotional expectations on life satisfaction. A positive b_1 suggests that higher emotional expectations are associated with higher life satisfaction, and vice versa.
3. b_2 **- Linear Effect of Emotional Experiences:** The coefficient b_2 indicates the direct impact of emotional experiences on life satisfaction. Similar to b_1, a positive b_2 implies that more frequent or intense emotional experiences correspond to greater life satisfaction.
4. b_3 **- Quadratic Effect of Emotional Expectations:** This coefficient captures the curvature effect of emotional expectations on life satisfaction. It represents how the relation between expectations and life satisfaction changes as expectations increase.
5. b_4 **- Interaction Effect:** The coefficient b_4 reflects the combined effect of emotional expectations and experiences. It shows how the relation between one variable and life satisfaction changes at different levels of the other variable.
6. b_5 **- Quadratic Effect of Emotional Experiences:** Similar to b_3, b_5 measures the curvature effect of emotional experiences on life satisfaction, indicating how this relation evolves as experiences intensify.

For the positive emotions, the baseline level of life satisfaction is $b_0 = 2.869$ ($SE = 0.079$, $p < .001$). This Intercept indicates a moderate baseline life satisfaction when emotional expectations and experiences are zero. The effect of emotional expectations on life satisfaction is minor and not statistically significant ($b_1 = -0.010$, $SE = 0.035$, $p = .772$), suggesting expectations alone do not strongly influence life satisfaction. There is a significant positive relation between the experience of positive emotions and life satisfaction ($b_2 = 0.382$, $SE = 0.050$, $p < .001$), highlighting the importance of actual emotional experiences. The quadratic effect is negligible ($b_3 = -0.001$, $SE = 0.013$, $p = .959$), indicating a nearly linear relation between expectations and life satisfaction. The interaction between expectations and experiences is significant ($b_4 = 0.039$, $SE = 0.016$, $p = .014$), suggesting a combined influence on life satisfaction. A slight, non-significant curvilinear relation is suggested between the experience of positive emotions and life satisfaction ($b_5 = -0.027$, $SE = 0.020$, $p = .175$).

For the negative emotions, the baseline life satisfaction when both expectations and experiences of negative emotions are zero is $b_0 = 2.190$ ($SE = 0.118$, $p < .001$). This suggests a lower baseline life satisfaction compared to positive emotions. The direct effect of expectations for negative emotions on life satisfaction is $b_1 = 0.080$ ($SE = 0.061$, $p = .193$), which is not statistically significant, indicating a weak influence. The coefficient $b_2 = -0.168$ ($SE = 0.067$, $p = .012$) shows a significant negative relation, implying that more frequent negative emotions are associated with lower life satisfaction. The significant quadratic effect ($b_3 = -0.039$, $SE = 0.017$, $p = .024$) suggests a non-linear relation, indicating a complex dynamic between expectations of negative emotions and life satisfaction. The coefficient $b_4 = 0.042$ ($SE = 0.022$, $p = .055$) suggests a marginally significant interaction effect, indicating the combined influence of expectations and experiences on life satisfaction. Last but not least, a significant positive quadratic effect ($b_5 = 0.063$, $SE = 0.013$, $p < .001$) suggests an increasing relation between the intensity of negative emotional experiences and life satisfaction, but with diminishing returns.

In summary, for positive emotions, the actual experience of these emotions is a significant determinant of life satisfaction, while the expectation of experiencing positive emotions plays a less substantial role. The interaction between these factors, though modest, is also significant, pointing to a nuanced interplay in influencing well-being. For negative emotions, the experience of negative emotions plays a

significant role in determining life satisfaction. The interaction and quadratic effects suggest a more complex relation compared to positive emotions, highlighting the multifaceted nature of emotional experiences and expectations in shaping well-being.

3 SURFACE PARAMETERS

The RSA, response surface analysis, aids in interpreting these interactions by examining the surface shape formed by these polynomial terms. Specifically, we analyze the line of congruence (LOC) and line of incongruence (LOIC) to understand how the alignment or misalignment of emotional experiences and expectations impacts life satisfaction.

The surface parameters in RSA, derived from the regression coefficients of our model, are critical for understanding the interaction effects and nonlinear relations between variables, providing a more comprehensive insight into the dynamics of emotional experiences and expectations in relation to life satisfaction. These parameters enable us to visualize and interpret the shape and contour of the response surface. By analyzing these surfaces, we can gain insights into whether congruence or incongruence between emotional experiences and expectations positively or negatively influences life satisfaction. This deeper analysis is vital in psychological studies where interactions are often more complex than simple linear relations. Below, we provide a detailed explanation of these surface coefficients:

1. p_{10} and p_{11} - **Position of First Principal Axis**: These coefficients define the orientation of the first principal axis on the RSA plot. The first principal axis, often described as the surface's "ridge," is projected onto the XY plane to understand the relation between the variables.
2. a_1 and a_2 - **Slope and Curvature along the Line of Congruence (LOC)**: These coefficients describe the nature of the relation along the LOC, which represents points where the level of emotional experience matches the level of expectations. The a_1, calculated as $a1 = b1 + b2$, indicates the slope or the direction of the relation, while a_2, calculated as $a2 = b3 + b4 + b5$, gives the curvature, showing how the relation bends along the LOC.
3. a_3 and a_4 - **Slope and Curvature along the Line of Incongruence (LOIC)**: These parameters explain the relation when there is a mismatch between emotional experiences and expectations. The a_3, calculated as $a3 = b1 - b2$, describes the slope along the LOIC, and a_4, calculated as $a4 = b3 - b4 + b5$, provides information about the curvature, offering insights into how life satisfaction varies with increasing incongruence between experience and expectation.

For the positive emotions, the significant linear additive effect ($a_1 = 0.372$, $SE = 0.061$, $p < .001$) suggests a strong linear relation along the line of congruence. This indicates that congruent levels of expectations and experiences of positive emotions have a pronounced effect on life satisfaction. The curvature effect ($a_2 = 0.011$, $SE = 0.027$, $p = .684$) is not significant, indicating a predominantly linear relation along the line of congruence without notable bending. A significant negative coefficient ($a_3 = -0.392$, $SE = 0.060$, $p < .001$) suggests that the direction of incongruence between experiences and expectations matters: life satisfaction is lower when one experiences low levels of emotions but the expectations for positive emotions are high compared to when one experiences high emotions but the norm/expectation is low. The significant curvature ($a_4 = -0.067$, $SE = 0.024$, $p = .004$) along the line of incongruence suggests a complex relation between incongruent levels of expectations and experiences and life satisfaction.

For the negative emotions, the analysis shows no significant linear additive effect ($a_1 = -0.088$, $SE = 0.094$, $p = .352$) along the line of congruence for negative emotions, suggesting a less pronounced linear relation between congruent levels of expectations and experiences. A significant curvature is observed ($a_2 = 0.066$, $SE = 0.027$, $p = .015$), indicating a nonlinear relation along the line of congruence. This suggests that the effect of congruence on life satisfaction is not uniformly linear. A significant shift away from the line of congruence ($a_3 = 0.247$, $SE = 0.087$, $p = .004$) indicates that higher life satisfaction is associated with levels of incongruence between low expectations and high experiences of negative emotions than the levels of incongruence between high expectations and low experiences of negative emotions . The analysis does not indicate significant curvature on the line of incongruence ($a_4 = -0.019$, $SE = 0.033$, $p = .578$), suggesting a more linear relation in this context.

4 CONCLUSION

In this Supplementary Material, we have described the application of Response Surface Analysis (RSA) in our research. Our study, structured as a brief report, necessarily condenses complex analyses. Therefore, this document serves as a transparent and detailed explanation, ensuring that the main text can be comprehensively understood. The clarity provided here, we hope, will aid readers in grasping the nuanced findings of our research, showcasing how RSA can unravel the multifaceted nature of emotional dynamics in psychological studies.

For a more detailed tutorial and guidelines on conducting and interpreting RSA, Humberg et al. (2019) offers step-by-step instructions, a checklist, and clarifications for testing congruence hypotheses. Additionally, Schönbrodt et al. (2018) provides detailed descriptions and formulas with examples and Barranti et al. (2017) provides a tutorial on how to test questions about similarity in personality and social psychology research, including a description and empirical demonstration of RSA.

REFERENCES

Barranti, M., Carlson, E. N., and Côté, S. (2017). How to test questions about similarity in personality and social psychology research: Description and empirical demonstration of response surface analysis. *Social Psychological and Personality Science* 8, 465–475. doi:10.1177/1948550617698204

Humberg, S., Nestler, S., and Back, M. D. (2019). Response surface analysis in personality and social psychology: Checklist and clarifications for the case of congruence hypotheses. *Social Psychological and Personality Science* 10, 409–419. doi:10.1177/1948550618757600

Schönbrodt, F. D., Humberg, S., and Nestler, S. (2018). Testing similarity effects with dyadic response surface analysis. *European Journal of Personality* 32, 627–641. doi:10.1002/per.2169
